# Upside-Down Preference in the Forskolin-Induced In Vitro Differentiation of 50B11 Sensory Neurons: A Morphological Investigation by Label-Free Non-Linear Microscopy

**DOI:** 10.3390/ijms24098354

**Published:** 2023-05-06

**Authors:** Luisa Zupin, Sotiris Psilodimitrakopoulos, Fulvio Celsi, Lina Papadimitriou, Anthi Ranella, Sergio Crovella, Giuseppe Ricci, Emmanuel Stratakis, Lorella Pascolo

**Affiliations:** 1 Institute for Maternal and Child Health IRCCS Burlo Garofolo, 34137 Trieste, Italy; 2 Institute of Electronic Structure and Laser, Foundation for Research and Technology—Hellas, 70013 Heraklion, Crete, Greece; 3 Biological Science Program, Department of Biological and Environmental Sciences, College of Arts and Sciences, University of Qatar, Doha 2713, Qatar; 4 Department of Medicine, Surgery and Health Sciences, University of Trieste, 34129 Trieste, Italy; 5 Department of Physics, School of Sciences and Engineering, University of Crete, 71003 Heraklion, Crete, Greece

**Keywords:** sensory neurons, second harmonic generation, multi-photon autofluorescence

## Abstract

In this study, we revealed a peculiar morphological feature of 50B11 nociceptive sensory neurons in in vitro culture related to the forskolin-induced differentiation of these cells growing upside-down on cover glass supports. Multi-photon non-linear microscopy was applied to monitor increased neurite arborization and elongation. Under live and unstained conditions, second harmonic generation (SHG) microscopy could monitor microtubule organization inside the cells while also correlating with the detection of cellular multi-photon autofluorescence, probably derived from mitochondria metabolites. Although the differentiated cells of each compartment did not differ significantly in tubulin or multi-photon autofluorescence contents, the upturned neurons were more elongated, presenting a higher length/width cellular ratio and longer neurites, indicative of differentiated cells. SHG originating from the axons’ microtubules represented a proper tool to study neurons’ inverted culture in live conditions without exogenous staining. This work represents the first instance of examining neuronal cell lines growing and differentiated in an upside-down orientation, allowing a possible improvement of 50B11 as a model in physiology studies of sensory neurons in peripheric nervous system disease (e.g., Fabry disease, Friedreich ataxia, Charcot–Marie–Tooth, porphyria, type 1 diabetes, Guillain–Barré syndrome in children) and analgesic drug screening.

## 1. Introduction

Studies performed on sensory neurons are a challenging task; researchers have employed primary dorsal root ganglion (DRG) neurons from rodents, but this approach is time-consuming and ethically controversial due to the high numbers of euthanized animals needed to obtain a good yield of cells for the subsequent experiments. Nevertheless, sensory neurons represent an indispensable tool to screen analgesic drugs or regenerative strategies, being the first preclinical step towards clinical trials for treating adult and pediatric subjects. Moreover, study of the signaling and physiology of sensory neurons is fundamental for research on and treatment of different pathologies involving the peripheric nervous system, such as Fabry disease [1], Friedreich ataxia [2], Charcot–Marie–Tooth [3], porphyria [4], type 1 diabetes [4,5], and Guillain–Barré syndrome [6] in children.

A possible alternative to dissociated DRG neurons is the employment of immortalized cell lines, such as the hybrid F11, ND7/23, and ND-C cell lines generated from the fusion of rat DRG neurons and mouse neuroblastoma cells or mouse MED17.11, human HD10.6, and rat 50B11 [7].

50B11 [8], an immortalized rat dorsal root ganglion sensory neuronal cell line, that, upon differentiation with forskolin, acquires typical sensory neuron morphology with extended neurites and markers of small-diameter nociceptive neurons, such as neurofilament and β tubulin III, represents a good model for mimicking nociceptive sensory neurons in vitro. Indeed, 50B11 has already been employed to study in vitro the effect of analgesic treatment [9], neuronal regeneration [10], neurotoxicity [11], and virus infection [12].

During the culturing of this cell line, we observed that 50B11 cells were able to grow and differentiate (upon forskolin treatment) not only in conventional conditions but also when the cells were allowed to grow on the underside of cover glasses deposited in multi-well culture plates in a condition of inverted gravitational orientation. Previous works have shown that primary neuronal cells can differentiate more and constitute complex neuronal networks when cultured in inverted conditions [13].

To study this peculiar cell differentiation type in 50B11 cells, we decided to approach live imaging techniques that do not require extensive sample processing and may offer unique biological information. Optical microscopy is widely used for long-lasting high-resolution visualization of living neural cultures. Conventional linear fluorescence microscopy (e.g., confocal microscopy) in combination with specific stains provides visualization of dynamic processes in vitro, with subcellular resolution, but suffers from possible out-of-focus photobleaching or photodamage and low penetration depths due to light scattering. Although in confocal microscopy z-plane separation is achieved by pinhole blocking the out-of-focus fluorescence (and 3D resolution is achieved), there is possible out-of-focus photobleaching or photodamage because the fluorescence originates from a wide excitation volume around the laser’s focal point in the sample [14]. Various nonlinear laser raster-scanning microscopy forms circumvent these limitations, including two-photon excited fluorescence (2pF) imaging [15]. Because in 2pF we have the participation of two photons, the nonlinear fluorescence signal decays rapidly out of focus, providing intrinsic axial resolution, eliminating the need for a confocal pinhole and restricting possible photobleaching or photodamage to a small signal volume around the focal point. The long (IR) illumination wavelengths allow deep imaging depths (~1 mm) [16]. Nevertheless, the markers utilized might disrupt the fragile structures of biological samples (i.e., neurons). A fluorescent molecule must be inserted into the system or produced by gene expression to be “visible” for 2pF imaging, which makes the whole process invasive [17]. Label-free SHG microscopy is based on a nonlinear process of light emission when the sample is illuminated with high-peak-power fs IR lasers. SHG is based on the non-linear property of intense light to combine two photons of the same energy in one of double energy. This unique property of light offers several advantages when used in optical microscopy: (a) Because the nonlinear signal decays rapidly out of focus, like in 2pF, SHG offers intrinsic axial resolution without needing a confocal pinhole, allowing 3D imaging. The possible photodamage is limited only in the tiny SHG active volume around the laser-focused spot, enabling minimally invasive imaging. (b) Owing to the long IR illumination wavelengths used, light scatters less. It can penetrate deeper into the samples, allowing profound penetration depths (imaging up to ~1 mm). (c) Due to its energy-conserving nature (elastic scattering of two non-resonant photons to one of double energy), there is no energy loss and no energy deposition onto the interacted sample. SHG is reasonably considered a non-invasive microscopy technique. (d) It is label-free for microtubules. Indeed, SHG only needs non-centrosymmetric structures and not resonant excitation photons (like in fluorescence) to occur, and microtubules offer this advantage [18]. The α-β dimers in the axons’ microtubules exhibit the non-centrosymmetric structure required to produce SHG. It has been reported that, in isolated or cultured live neurons, SHG signals derive from microtubules in neurites, especially when they are in an ordered, GTP-bound state, and the signal intensity increases toward the axonal end (growth cone) [19,20]. The microtubule network is an active part of the cytoskeleton. In most laboratories, it can be monitored only with staining, which usually employs a post-fixation immunofluorescence antibody-based imaging technique or labelling using taxol or derivatives that partially block the microtubules’ assembly, or with the production of exogenous plasmids encoding for marked microtubules [21]. Aβ-tubulin dimers form microtubules under “dynamic instability,” where the fibrillary microtubule structures continually grow and shrink, never acquiring a steady-state conformation [22]. Thus, analyzing microtubules in live cells is challenging, and SHG can overcome this limitation. When fs IR laser light interacts with the non-centrosymmetric microtubules in axons, two off-resonant photons (that are not absorbed) are combined and detectable SHG is generated, providing a unique label-free source of contrast that reflects the microtubules’ content and structure and intrinsic factors of the macromolecules, such as the number, density and arrangement of the microtubules [19]. In addition, when fs IR laser light interacts with living unlabeled neural cultures, multi-photon autofluorescence can be monitored. A comparison of the advantages and disadvantages of each imaging technique can be found in work by Pallen et al. [23]. The endogenous molecules that provide label-free multi-photon fluorescence signals in neuronal cultures are FAD and NADH [24,25]. Endogenous multi-photon fluorescence in living cells originating from NADH has already been explored to reveal the mitochondria [26]. Therefore, the target molecules for multi-photon autofluorescence are FAD and NADH. 

In the present work, we took advantage of the properties of label-free non-linear microscopy (combined SHG and multi-photon autofluorescence) to monitor the different growths and differentiations of 50B11 cells on the upper and bottom sides (normal and inverted gravitational orientations) of glass substrates, comparing different time points (24, 48, and 72 h).

## 2. Results

The growth of more than three different preparations of 50B11 cells was monitored for 3 days after forskolin addition (75 μM), with analysis of the cells on the topside and underside of the glass substrates of the same wells. SHG microscopy was exploited, coupled with the endogenous multi-photon autofluorescence of the cells, employed to define the cell border and the specificity of the SHG signals on cellular structures to monitor cell differentiation. Although a clear difference in SHG or multi-photon autofluorescence was not found between the cells growing on the two sides of the cover glasses, the morphology of the cells growing on the topside appeared to include a high percentage with rounded bodies and short neurites, with a progressive increase in differentiating cell number during the time course. Conversely, in the same well, the cells growing underside appeared to differentiate earlier, quickly producing a highly enlarged neural network, still increasing from day 1 to day 3, when all the cells were particularly elongated.

**Figure 1:**
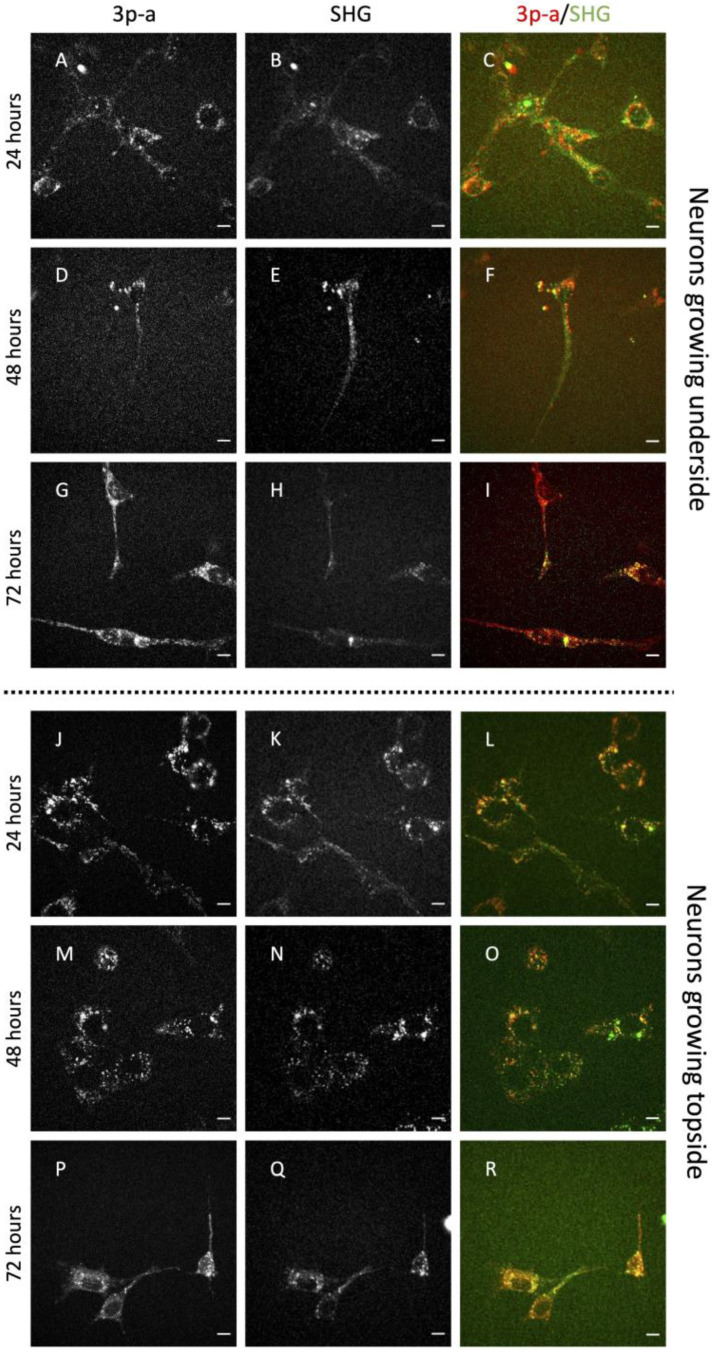
Multi-photon autofluorescence (3p-a) signal (in red) and second harmonic generation (SHG) (in green) at 24, 48, and 72 h in cells growing on the underside and topside of cover glass substrates. Both single channels and the merge are displayed. Scale bars show 10 μm. Cells growing underside: 24 h—(**A**) 3p-a, (**B**) SHG, (**C**) merge; 48 h—(**D**) 3p-a, (**E**) SHG, (**F**) merge; 72 h—(**G**) 3p-a, (**H**) SHG, (**I**) merge; Cells growing topside: 24 h—(**J**) 3p-a; (**K**) SHG, (**L**) merge; 48 h—(**M**) 3p-a, (**N**) SHG, (**O**) merge; 72 h—(**P**) 3p-a, (**Q**) SHG, (**R**) merge.

A strong SHG signal was detected during the time course (i.e., 24, 48 and 72 h) in both cell populations growing on the underside and topside of the cover glasses (Figure 1, SHG is displayed in green). The multi-photon autofluorescence of the cells was also detected, and this was employed in order to focus on and identify the cells and to determine that the cells under analysis were viable and functional (Figure 1; multi-photon autofluorescence is displayed in red). By employing 3D multi-photon autofluorescence reconstruction (z-stack of 180 μm, step 2 μm), it is also possible to see the two sides of the glass (in the middle) where the cultured neurons were grown (Supplementary Video S1).

**Figure 2:**
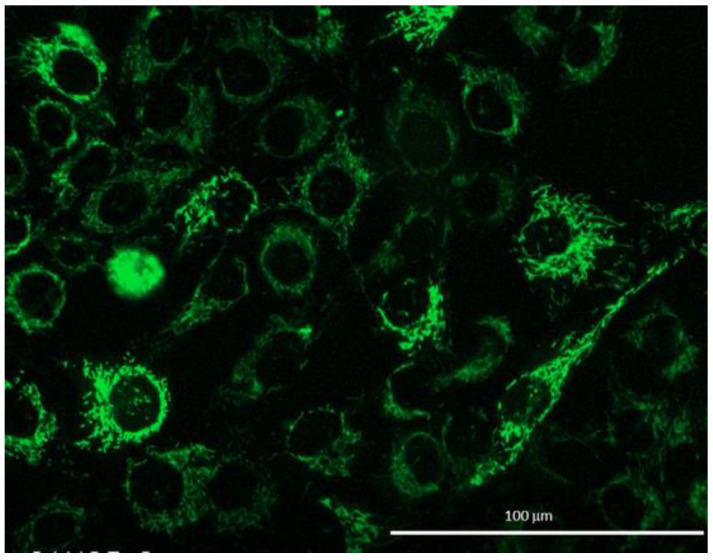
Mitochondria staining in the 50B11 cells (Mitotracker Green fluorescence). Scale bar: 100 µm.

Indeed, these signals were estimated to derive from the reduced forms of NADH and FAD within the mitochondria [27]. A few samples of 50B11 cells differentiated on the topside of the glass to confirm this observation support this; the mitochondria were stained live with the Mitotracker probe, and the pattern of mitochondrial fluorescent signals (Figure 2) was similar to that of multi-photon autofluorescence.

Nevertheless, some differences can be observed, probably due to the two methods employed; Mitotracker staining is based on the mitochondrial membrane potential-dependent accumulation of the dye inside mitochondria in live cells, which links to the thiol groups of mitochondrial proteins. Therefore, the signals appear extended, showing the classical mitochondrial morphology [28]. On the other hand, multi-photon autofluorescence probably derives from the excitation of NADH and FAD molecules not evenly spaced in the mitochondria. It is more localized and has a spot-like appearance [27]. The lateral resolution of the multi-photon autofluorescence and SHG images presented here (using the 40×, 1.3NA objective and 1030 nm excitation wavelength) is ~1 μm.

Mitochondrial movements were also recorded with multi-photon autofluorescence and with Mitotracker staining to assess if fusion/fission processes characteristic of mitochondria dynamics [27] occurred: a similar mitochondria velocity was registered (Supplementary Video S2 for Mitotracker staining and Supplementary Video S3 for multi-photon autofluorescence).

**Figure 3:**
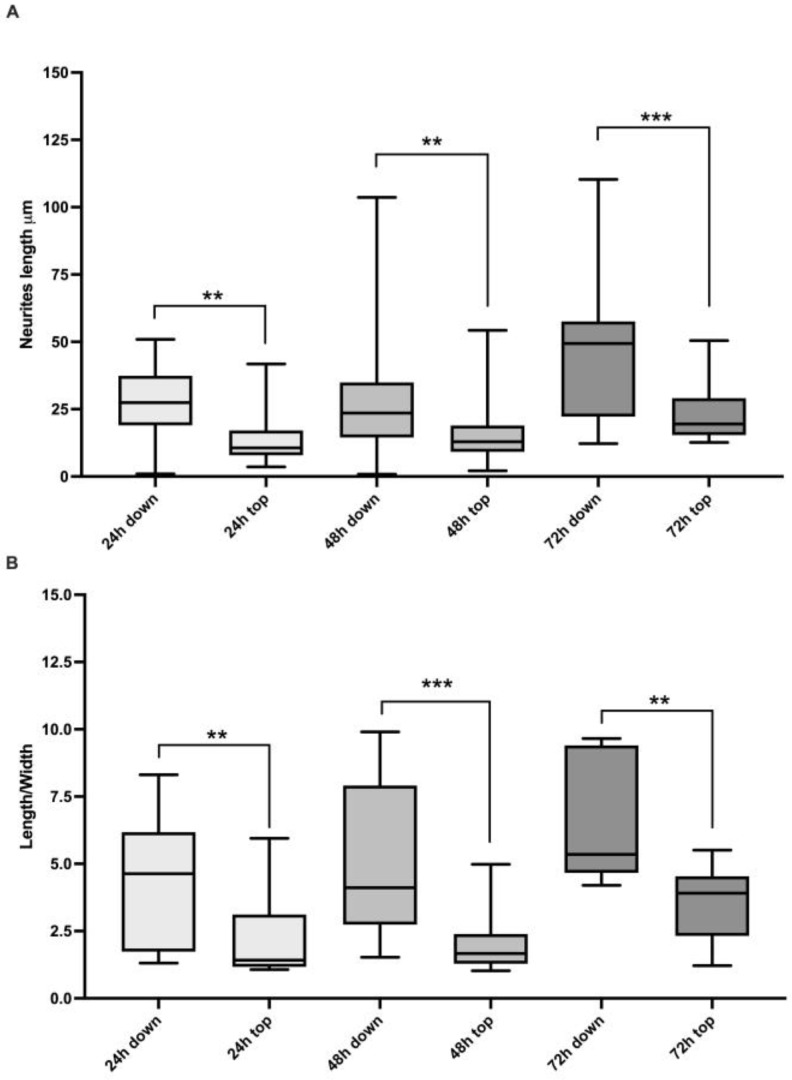
The lengths of neurites in μm (**A**) displayed as median ± standard deviation) and the length/width ratios (**B**) during the time course in the cells growing on top of and underside cover glasses. Results from the Mann–Whitney test are shown: ** *p*-value < 0.01, *** *p*-value < 0.001.

To confirm the morphological observations, we measured the neuronal features. Although there was variability between the two cell populations, the length of the neurites and length/width ratio were significantly higher in cells developing on the underside than those growing topside at the different time points of the time course (neurite length Mann–Whitney tests: 24 h, *p*-value = 0.003; 48 h *p*-value = 0.007; 72 h *p*-value = 0.0008; Figure 3A; length/width ratio Mann–Whitney tests: 24 h, *p*-value = 0.005; 48 h *p*-value = 0.0001; 72 h *p*-value = 0.005; Figure 3B). 

Indeed, considering the fraction of the cells with an elongated appearance (when the longer dimension was at least four times longer than the short dimension), the cells growing underside showed higher percentages of elongated cells, being 52%, 50%, and 100% at 24, 48, and 72 h, respectively, while in the neuronal population proliferating topside 21%, 6%, and 50% of the cells were elongated (Table 1).

## 3. Discussion

In this study, rat immortalized dorsal root ganglion sensory neuronal cell line 50B11, differentiated by using forskolin, was employed as a model of nociceptive sensory neurons, and its growth was observed on glass substrates. These 50B11 cells can attach and grow on top of and under the cover glass, with different features on the two sides. Intriguingly, while on the top the cells exhibit a more roundish shape, resembling a fibroblast-like appearance with clustered growth of numerous cells, on the bottom side, they create a neural network with elongated protrusions and globose soma, better representing neuronal features.

The close relation between cells and substrate is crucial, particularly for differentiating neuronal cells [29]; previous evidence shows that proper attachment involving microfilaments is necessary to stabilize the growth cone margin and the extension of nerve fibers [30].

50B11 binding on the underside of cover glasses is weak; therefore, to overcome the classical procedure of staining and fixation, SHG microscopy was exploited, a technique that allows the detection of the SHG signal from microtubules in live conditions and without staining [19,20].

The cells were monitored for 3 days, showing an increment in differentiation during the time course. Indeed, 50B11 showed neurite elongation that was particularly evident at 72 h post differentiation when, in all the cells growing underside, the length/width ratio exceeded 4. In contrast, in the cell population on the top side, the fraction of elongated cells was 50%. A 3D reconstruction of the cells growing on the cover glasses’ sides confirmed the presence of two different cell populations in the two microscopy plans. SHG was fundamental to observe the cells’ and microtubules’ presence. However, no specific differences in SHG signal (derived from microtubule content and organization) were seen between the cells growing at the top and underside of the substrate. This lack of difference could be expected, since the microtubules constitute all cells’ components and are present in a large amount without distinction between cellular types [31].

Intriguingly, with multi-photon microscopy, we could also monitor the multi-photon autofluorescence of the cells, probably derived from NADH and FAD, whose presence is higher in mitochondria than cytosol [27]. Indeed, when illuminating cells with the 1030 nm wavelength, by exploiting 3p absorption, NADH and FAD were excited by 343 nm, and their fluorescence emission was detected at 620 ± 26 nm [32]. Therefore, multi-photon autofluorescence monitoring over time allowed us to define a movement of the signals along the neurites of the cells, most likely derived from the fusion/fission mitochondrial network dynamics. The specificity for mitochondria of the multi-photon autofluorescence signals detected was confirmed by comparison with experiments employing a tracker for mitochondria that showed a similar pattern of conventional light fluorescence in these cells and also a comparable velocity in these organelle movements. 

The signals from multi-photon autofluorescence and SHG co-localize in some regions, indicating that the mitochondria are actively transported within the microtubule network [33].

Neuritogenesis provokes the remodeling of the cytoskeleton, involving actin and microtubules, and promotes cone growth development with cellular structure reorganization and reorientation [34]. The most impressive feature we noted in our experiments is the peculiar morphological appearance of the cells growing under the supports, even if it is relatively common to observe cells attached under the glass slides. Indeed, the growth of cells under the supports was previously observed using the inverted cell culture [35], transwell assay [36], and ceiling culture methods [37]. Moreover, in accordance with our observations, the inverted culture method was also previously employed to grow mouse primary hippocampal neurons showing an improved neurogenesis process and an increment in cell survival in this condition. The authors speculated that low oxygen levels and an increment in trophic factors in the confined microenvironment supported better neuron development [13], and a similar supposition can apply to our sensory neuron model. Notably, we did not register an improvement in cell survival in the upside-down growing condition; on the contrary, 50B11 cells seemed to be less adhesive to the surface, thus making the live microscopy setting employed here an optimal approach to studying their delicate differentiation.

Notably, the approach described here, i.e., the combination of two sensitive laser techniques under a single microscope, SHG and multi-photon autofluorescence, is expected to also improve the study of other sensible neuronal and primary cells in a label-free modality, translating the results obtained in 50B11 cells to other possible neuroscientific applications. 

This technique could also be decisive for studying diseases or conditions where the tubulin dimers are lost, representing a tool to monitor tubulin modification vs control conditions [19] while enabling monitoring mitochondrial functionality [38].

## 4. Conclusions

The live study of neuronal microtubule configurations is a tremendous challenge for neuroscientists. This work demonstrated that SHG can be successfully applied to a sensory neuron cell model to monitor real-time living cells. The coupling of SHG with multi-photon autofluorescence, after optimal selection of the optical set-up, was essential to focus on the cells and identify the specificity of the nonlinear signals detected, which, when colocalized, can indicate the active dynamics of mitochondria transport within the microtubule network [33]. Our final aim, i.e., label-free comparison of the live 50B11 cells growing on the two sides of the silicon substrates, was achieved thanks to the exploitation of this combined strategy.

Moreover, the assessment of different neuronal differentiations was also possible by evaluating neurite length and elongated cells, thus confirming our morphological observations with functional data representative of neuronal development.

These observations are in line with what was previously seen in primary hippocampal cultures, where cells were seeded on the cover glass and turned upside-down after attachment. These “upturned” cells display increased neurite length and the formation of complex networks; authors have suggested a possible physiological mechanism in which these cells experience a reduced-oxygen environment and increased trophic support. Our model possibly undergoes similar conditions, promoting neuronal differentiation. 

With this work, we add information to better characterize the growth of the 50B11 cell line, corroborating its employment as a model for different applications in neuroscience research [7]. Indeed, this cell line is a valuable alternative to primary DRG neurons and can be exploited for the screening of analgesic and regenerative drug responses, a first step toward the long process of a drug being approved for clinical use in both adult and pediatric subjects or in the study of peripheral sensory neuron pathologies such as Fabry disease [1], Friedreich ataxia [2], Charcot–Marie–Tooth [3], porphyria [4], type 1 diabetes [4,5], and Guillain–Barré syndrome [6] in children.

## 5. Materials and Methods

### 5.1. 50B11 Cell Line

The 50B11 immortalized rat dorsal root ganglion sensory neuronal cell line was employed as a model of sensory neurons [8]. The cells were maintained in neurobasal medium (21103049, Life Technologies, Thermo Fisher Scientific, Waltham, MA, USA) supplemented with 10% fetal bovine serum (Euroclone, Pero, Milan, Italy), 2% B27 (Life Technologies), 0.22% glucose (Sigma-Aldrich, Saint Louis, MO, USA), 0.2 mM glutamine (Euroclone), and 100 U/mL penicillin/streptomycin (Euroclone, Pero, Milan, Italy). The cells were seeded at a cellular density of 20,000 cells per well in glass coverslip #1 in 24 multi-well plates. After 24 h from the initial seeding, the cells were treated with forskolin (75 μM, F6886, Sigma-Aldrich) to induce differentiation and neurite sprouting, a process visible after 24 h of treatment, and the medium with forskolin was changed every 24 h. At 24, 48, or 72 h, the cells were analyzed with the techniques described below.

### 5.2. Custom-Build Multi-Photon Microscope

Although 2pF and SHG microscopies are based on fundamentally different phenomena, they can be combined in the same instrument, providing complementary information. Our custom-built 2p microscope is capable of recording 2pF and SHG signals. It is based on an Axio observer Z1 microscope (Zeiss, Oberkochen, Germany) and an fs laser (FLINT, Light Conversion, Vilnius, Lithuania) centred at 1030 nm, 80 MHz, 30 fs. The laser passes through a pair of galvanometric mirrors (which move accordingly to create a raster scanning scheme). It is tightly focused onto the sample after passing a high-numerical-aperture objective lens (40× 1.3NA). The microscope has two output ports, one for detecting 3pF and one for SHG. Appropriate filters have been placed in front of the detectors to eliminate any unwanted signals from detection (680SP for cutting the laser, bandpasses of 514/3 nm and 620/52 nm for SHG and 2pF, respectively—Semrock, NY, USA). The detector module is based on a photomultiplier tube (PMT) (Hamamatsu, Japan), transforming light into voltage values. While the laser beam raster scans the sample, the PMT records values at frequent intervals. These values are then transformed into a matrix and a contrasted image. The process lasts ~1 s for an image of 500 × 500 pixels/measurements (pixel dwell time ~6 μs), while the field of view using the 40× objective is ~160 μm with a ~0.5 μm radial resolution. An analytical description of the setup can be found in Psilodimitrakopoulos et al. [39]. Here, both multi-photon fluorescence and SHG are detected sequentially in the reflection mode. In all the experiments, the mean laser power was kept constant at the sample plane at ~70 mW. 

In usual fluorescence (like in confocal microscopy), one photon of appropriate energy is absorbed and promotes an electron to one of the excited energy states of a molecule. Then, the electron returns to the ground state radiatively by emitting one fluorescence photon. In 2p-excited fluorescence, two photons of lower energy (longer wavelength) are absorbed and promote the electron to the same excited state as before. Then, the electron returns to the ground state radiatively by emitting again one fluorescence photon. In 3p-excited fluorescence, three photons of even lower energy (longer wavelength), are absorbed to promote the electron to the same excited state. Then, like before, the electron returns to the ground state radiatively by emitting again one fluorescence photon. For the 1030 nm excitation wavelength, the photons do not have enough energy to be absorbed by NADH or FAD [32]. In 2p absorption, two photons of 1030 nm act like the absorption of one photon at 515 nm, which is not within the absorption spectra of FAD or NADH. In 3p absorption, three photons of 1030 nm act like the absorption of one photon at 343 nm, which is within the absorption spectra of both NADH and FAD [32]. Independently of the excitation wavelength (515 nm for 2p absorption of 1030 nm and 343 nm for 3p absorption of 1030 nm), the fluorescence emission spectra of NADH and FAD will be the same as in one photon absorption process. Thus, taking into account the NADH and FAD absorption and emission spectra seen in [32], we conclude that with 1030 nm we excite both NADH and FAD using 3p absorption. By choosing to detect in the range of 620 ± 26 nm, we collect the fluorescence from both NADH and FAD [32]. Additionally, by using the 620/52 nm range, we are far away from the 514 ± 1.5 nm where the SHG is detected, separating in this way more efficiently the detection of the nonlinear signals.

### 5.3. Mitochondrial Morphology

Mitochondrial presence and features were assessed using Mitotracker Green labelling (M7514, Thermo Fisher Scientific). The cells were stained with Mitotracker Green (10 µM) in Hanks’ Balanced Salt Solution (HBSS) for 30′ at 37 °C. Then, the cells were washed three times in HBSS, maintained in complete neurobasal medium without phenol red, and observed with the Cytation 5 Cell Imaging Multi-Mode Reader (Biotek, Winooski, VT, USA). The cells were monitored for 30′, and images were taken every 1 min with the z-stack imaging mode (10 µm of thickness) at 20× magnification. Then, the mitochondrial movement was reconstructed employing Gen 5 software (Biotek). This study’s multi-photon excitation of Mitotracker Green was at 515 nm from two photons of 1030 nm. 

### 5.4. Morphological Assessment under Microscopy

The measurements were performed using Image J software’s Neuron J plugin [40,41]. The lengths of the neurites and the length-to-width ratio were registered. The cell was defined as elongated when the longer dimension (length) was at least four times longer than the short dimension (width) [42].

### 5.5. Statistical Analysis

Comparison of neurites’ lengths and length/width ratios between the neurons growing on the topside and underside of the cover glasses at the three time points was evaluated with the Mann–Whitney test (two sides), utilizing R software [43].

## Figures and Tables

**Table 1:** The percentages of elongated cells and the total numbers of cells measured.

Condition	n. Analyzed Cells	% of Elongated Cells
24 h down	21	52%
24 h top	19	21%
48 h down	10	50%
48 h top	33	6%
72 h down	7	100%
72 h top	12	50%

## Data Availability

Data is contained within the article and Supplementary Material.

## Supplementary Materials

The following supporting information can be downloaded at: https://www.mdpi.com/article/10.3390/ijms24098354/s1.
